# Income inequality as a determinant of neonatal mortality in the Americas during 2000–2019: implications for the attainment of Sustainable Development Goal target 3.2

**DOI:** 10.1186/s12939-024-02157-9

**Published:** 2024-05-27

**Authors:** Antonio Sanhueza, Daniel A. Cueva, Oscar J. Mujica, Patricia Soliz, Pablo Duran

**Affiliations:** 1 Department of Evidence and Intelligence for Action in Health, Pan American Health Organization, PAHO/WHO, Washington, D.C USA; 2 Independent Consultant, Tarragona, Spain; 3 Latin American Center for Perinatology, Women’s Health, and Reproductive Health, CLAP/WR), Montevideo, Uruguay

**Keywords:** Neonatal mortality, Social determinants of Health, Americas, Sustainable development goals

## Abstract

**Background:**

The work of the WHO Commission on the Social Determinants of Health has been fundamental to provide a conceptual framework of the social determinants of health. Based on this framework, this study assesses the relationship of income inequality as a determinant of neonatal mortality in the Americas and relates it to the achievement of the Sustainable Development Goal target 3.2 (reduce neonatal mortality to at least as low as 12 deaths per 1,000 live births). The rationale is to evaluate if income inequality may be considered a social factor that influences neonatal mortality in the Americas.

**Methods:**

Yearly data from 35 countries in the Americas during 2000–2019 was collected. Data sources include the United Nations Inter-agency Group for Child Mortality Estimation for the neonatal mortality rate (measured as neonatal deaths per 1,000 live births) and the United Nations University World Institute for Development Economics Research for the Gini index (measured in a scale from 0 to 100). This is an ecological study that employs a linear regression model that relates the neonatal mortality rate (dependent variable) to the Gini index (independent variable), while controlling for other factors that influence neonatal mortality. Coefficient estimates and their robust standard errors were obtained using panel data techniques.

**Results:**

A positive relationship between income inequality and neonatal mortality is found in countries in the Americas during the period studied. In particular, the analysis suggests that a unit increase in a country’s Gini index during 2000–2019 is associated with a 0.27 (95% CI [− 0.04, 0.57], *P* =.09) increase in the neonatal mortality rate.

**Conclusion:**

The analysis suggests that income inequality may be positively associated with the neonatal mortality rate in the Americas. Nonetheless, given the modest magnitude of the estimates and Gini values and trends during 2000–2019, the findings suggest a potential limited scope for redistributive policies to support reductions in neonatal mortality in the region. Thus, policies and interventions that address higher coverage and quality of services provided by national health systems and reductions in socio-economic inequalities in health are of utmost importance.

## Background

There have been important reductions in child mortality during the last two decades in the Americas. For instance, the under-five mortality rate, expressed as the number of under-five deaths per 1,000 live births, in the region decreased from 26 in 2000 to 13 in 2020, representing a 3.5% annual rate of reduction during the 2000–2020 period [1]. Regarding the neonatal mortality rate, expressed as the number of neonatal deaths per 1,000 live births, it decreased in the Americas from 13 in 2000 to 7 in 2020, representing a 3% annual reduction rate. Nonetheless, despite the progress in reducing child mortality in the region, the lives of almost 200 thousand under-five children were lost in 2020 [1]. From these deaths, 55% occurred during the neonatal period, which illustrates the importance of focusing on neonatal deaths to end child mortality [1].

To catalyze progress towards ending neonatal mortality, global initiatives including the Millennium Development Goals (MDGs), Sustainable Development Goals (SDGs), *Global Strategy for Women’s, Children’s and Adolescents’ Health* [2], the Every Newborn Action Plan [3], and regional initiatives, such as the *Plan of Action for Women’s, Children’s, and Adolescents’ Health* [4] and *Regional Strategy and Plan of Action for Neonatal Health within the Continuum of Maternal, Newborn, and Child Care* [5], have set specific objectives and targets to reduce child mortality in the Americas. Importantly, target 3.2 from the third Sustainable Development Goal (SDG 3) to ensure healthy lives and promote well-being for all at all ages aims to “reduce neonatal mortality to at least as low as 12 per 1,000 live births” by 2030 [6]. Based on the latest data, out of the 35 countries in the Americas, 7 countries currently have neonatal mortality rates above the SDG 3.2 target. Thus, progress in ending preventable child deaths during the current decade will be key to meet the SDG 3.2 target for 2030 in countries in the Americas.

The influence of socioeconomic factors and different types of inequalities on health outcomes has been an area of research with important advances during the last two decades. Particularly, the work of the WHO Commission on the Social Determinants of Health [7] has been fundamental to provide a conceptual framework of the social determinants of health. As a result, the global evidence base on the social determinants of health and health equity has expanded. Still, to the best of our knowledge, no analyses have examined the role of income inequality as a determinant of neonatal mortality in the Americas and assessed its implications for the attainment of the SDG target 3.2, which is the aim of this paper. Particularly, our research questions are: (i) Does income inequality influence the neonatal mortality rate in countries in the Americas? (ii) If so, what changes in income inequality would be related to achieving SDG target 3.2 in countries in the Americas with a neonatal mortality rate above 12 deaths per 1,000 live births? Our hypothesis is that income inequality and the neonatal mortality rate are positively associated, so that reductions in income inequality may result in reductions in the neonatal mortality rate, contributing to the achievement of SDG 3.2 in the Americas.

## Materials and methods

### Data and variables

The analysis focuses on 35 countries from the Americas during 2000–2019, with yearly country-level data available for each country. The countries studied are: Antigua and Barbuda, Argentina, Bahamas, Barbados, Belize, Bolivia, Brazil, Canada, Chile, Colombia, Costa Rica, Cuba, Dominica, the Dominican Republic, Ecuador, El Salvador, Grenada, Guatemala, Guyana, Haiti, Honduras, Jamaica, Mexico, Nicaragua, Panama, Paraguay, Peru, Saint Kitts and Nevis, Saint Lucia, Saint Vincent and the Grenadines, Suriname, Trinidad and Tobago, United States of America, Uruguay, and Venezuela.

The neonatal mortality data used in the analysis comes from the United Nations Inter-agency Group for Child Mortality Estimation (UN IGME) 2021 database [8]. The specific indicator generated by UN IGME is the mean estimate of the neonatal mortality rate. This indicator represents the probability of dying during the first 28 completed days of life, and is expressed as the number of neonatal deaths per 1,000 live births. It is calculated based on the yearly number of deaths of infants during their first 28 days of life and the yearly number of live births by country. All available nationally representative data are compiled annually from different data sources including civil registration systems, population censuses, and household surveys, with a preference for data from the first [9]. The quality of these data for all countries is assessed, excluding those data with considerable non-sampling errors or omissions [1]. Finally, a Bayesian hierarchical splines regression model [10] is applied to the data, which may account for random errors in sample surveys. This methodology relates the neonatal mortality rate to the under-five mortality rate and a country-specific effect, effectively capturing country-specific trends in neonatal mortality across time [10]. The results from the model are used for interpolation and extrapolation, resulting in a smoothed time-series of the neonatal mortality rate for each country with no gaps and up to a common year [9]. The application of this common methodology across countries allows for comparability of data between countries during a given year as well as within countries during different years, overcoming differences in underlying data sources [1].

The income inequality variable used in the analysis is the country’s Gini index from UNU-WIDER’s World Income Inequality Database (WIID) Companion 31 May 2021 version [11], which captures the distribution of net income per capita within a country. The scale of the Gini index used in the analysis ranges from a minimum of 0 (perfect equality) to 100 (perfect inequality), with higher values indicating greater income inequality.

A review of the literature was carried out to identify other variables that may influence neonatal mortality (control variables) and these were classified into three groups: (i) related to individuals, (ii) related to health systems and (iii) related to the socioeconomic and macroeconomic contexts [12–16]. The proportion of newborns with low birthweight from the UNICEF-WHO Low birthweight estimates [17] is used as the variable from group (i). The control variables used for group (ii) are the proportion of women aged 15–49 years who have their need for family planning satisfied with modern methods from the World Health Organization [18] and the proportion of births attended by skilled health personnel from the World Bank [19]. Finally, socioeconomic and macroeconomic variables from group (iii) are: the secondary school enrollment rate from the World Bank [19], logged Gross Domestic Product (GDP) per capita from the Penn World Table version 10.0 [20], and the expenditure in health as a percentage of GDP from the World Health Organization [21]. These control variables were selected after reviewing the literature on social determinants of the neonatal mortality rate.

### Estimating the role of income inequality as a determinant of neonatal mortality

The work of the WHO Commission on the Social Determinants of Health [7] is used as a starting point to conceptually relate income inequality to neonatal mortality. Our proposed linear regression model considers countries as the unit of analysis and years as the time periods and is the following:

1 $$ {y}_{it}=\beta {x}_{it}+\sum _{j=1}^{k}{\gamma }_{j}{c}_{jit}+{{\alpha }_{i}+{\lambda }_{t}+\epsilon }_{it} $$

, where $$ {y}_{it}$$ and $$ {x}_{it}$$ represent the neonatal mortality rate (dependent variable) and income inequality (independent variable) in country $$ i$$ during year $$ t$$, respectively. The model includes $$ k$$ control variables ($$ {c}_{1it},\dots, {c}_{kit} $$) that may influence the neonatal mortality rate. Additionally, the model controls for country-specific effects ($$ {\alpha }_{i}$$) and year-specific effects ($$ {\lambda }_{t}$$). The former account for country-specific time-invariant characteristics that may vary across countries, such as geography, climate, culture, and social norms; whereas the latter control for common time trends in neonatal mortality that are shared among countries. The coefficient estimate on income inequality ($$ \widehat{\beta }$$) in Eq. (1) characterizes the role of income inequality on the neonatal mortality rate. Finally, $$ {\epsilon }_{it}$$ is the idiosyncratic error that accounts for variation in the neonatal mortality rate that cannot be explained by the model.

### Estimating implications of changes in income inequality for the attainment of SDG 3.2

SDG target 3.2 aims to “reduce neonatal mortality to at least as low as 12 per 1,000 live births” by 2030 [6]. To estimate the change in income inequality that would result in achieving this target in each country $$ i$$ that, based on data available for the latest time period $$ \stackrel{-}{t}$$, has a neonatal mortality rate higher than 12 deaths per 1,000 live births, the following formula is used:

2 $$ \widehat{{\Delta }{x}_{i} }= \frac{{y}_{i,t=\stackrel{-}{t}}-12}{\widehat{\beta }} $$

, where $$ {y}_{i,t=\stackrel{-}{t}}$$ is the neonatal mortality rate for country $$ i$$ in year $$ \stackrel{-}{t}$$, 12 is the SDG 3.2 target, and $$ \widehat{\beta }$$ is the coefficient estimate from Eq. (1). This exercise may be interpreted as a hypothetical policy experiment in which there is an exogenous policy action to change the Gini index in the countries that have a neonatal mortality rate higher than 12 deaths per 1,000 live births whilst keeping other country characteristics fixed. In this setting, the results can be interpreted as the expected change in the Gini index, exclusively attributed to the policy, that would reduce the neonatal mortality rate to 12 deaths per 1,000 live births (the SDG 3.2 target for neonatal mortality).

### Computational implementation of statistical methods

Analyses are performed in Stata 15 [22]. The *xtivreg* command is used to estimate Eq. (1) via the Generalized Two Stage Least Squares (G2SLS) estimator proposed by Balestra and Varadharajan-Krishnakumar [23]. This instrumental variables approach addresses endogeneity concerns, such as confounding, as it decomposes income inequality into its component uncorrelated to the error term, so that exogenous variation in income inequality is used in the analysis. For the implementation of this estimation strategy, the second, third, and fourth lags of income inequality i.e., income inequality during the previous two, three, and four years, are used as instrumental variables for income inequality.

Country-specific effects are modelled as random effects, whereas time-specific effects are incorporated in the analysis by using year indicators. To account for the possibility of autocorrelation and heteroscedasticity, cluster robust standard errors are calculated, with countries as the cluster dimension. These account for heteroscedasticity and allow error terms to be arbitrarily correlated within each country, but assume they are uncorrelated across countries [24]. Finally, the delta method is applied to the estimates of Eq. (1) to estimate the mean change in inequality required to achieve SDG 3.2 and its corresponding confidence interval (Eq. 2).

## Results

### Descriptive statistics and data visualization

Summary statistics are reported in Table 1. There is full availability of data for the neonatal mortality rate and the Gini index during 2000–2019 for all 35 countries studied. The proportion of newborns with low birthweight and the secondary school enrollment rate are the variables with the least data availability for the 2000–2019 period. The mean neonatal mortality rate for the 35 countries studied during 2000–2019 is 11.95 deaths per 1,000 live births, with a minimum value of 2.31 and a maximum value of 30.58. Income inequality among the 35 countries during 2000–2019 is considerably high, with the Gini index ranging from 32.83 to 63.51 and a mean of 49.19.

**Table 1:** Summary statistics

Variable		Mean	Standard Deviation.	Min.	Max.	Observations
Neonatal mortality rate	overall	11.95	6.12	2.31	30.58	*N* = 700
	between		5.82	3.05	28.3	*n* = 35
	within		2.14	4.02	21.22	T = 20
Gini index	overall	49.19	5.93	32.83	63.51	*N* = 700
	between		5.48	33.6	60.15	*n* = 35
	within		2.44	39.85	60.4	T = 20
Logged GDP per capita (PPP)	overall	9.35	0.76	5.53	11.06	*N* = 680
	between		0.7	7.43	10.92	*n* = 34
	within		0.32	6.15	10.48	T = 20
Health expenditure (% of GDP)	overall	6.52	2.32	3.06	16.84	*N* = 700
	between		2.2	3.94	15.45	*n* = 35
	within		0.82	3.14	10.5	T = 20
Secondary school enrollment (%)	overall	73.16	14.46	24.5	99.81	*N* = 423
	between		14.46	38.83	94.35	*n* = 34
	within		6.29	52.32	92.16	T = 12.44
Low birth weight among newborns (%)	overall	9.88	2.76	5.18	16.26	*N* = 448
	between		2.8	5.41	15.88	*n* = 28
	within		0.23	9.26	10.64	T = 16
Skilled attendant at birth (%)	overall	94.39	10.84	23.8	100	*N* = 496
	between		13.7	35.48	99.92	*n* = 35
	within		4.36	69.65	118.81	T = 14.17
Family planning satisfied with modern methods (%)	overall	74.61	11.88	33.1	89.9	*N* = 660
	between		11.72	40.43	88.6	*n* = 33
	within		2.77	64.02	84.07	T = 20

**Fig. 1:**
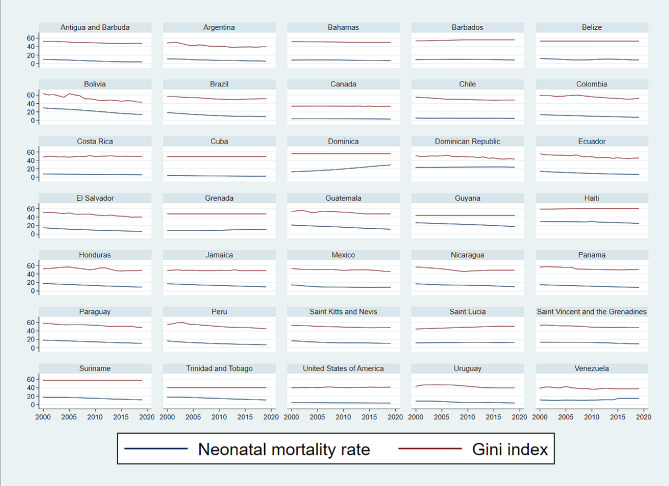
Neonatal mortality rate and Gini index for countries of the Americas, 2000–2019

**Fig. 2:**
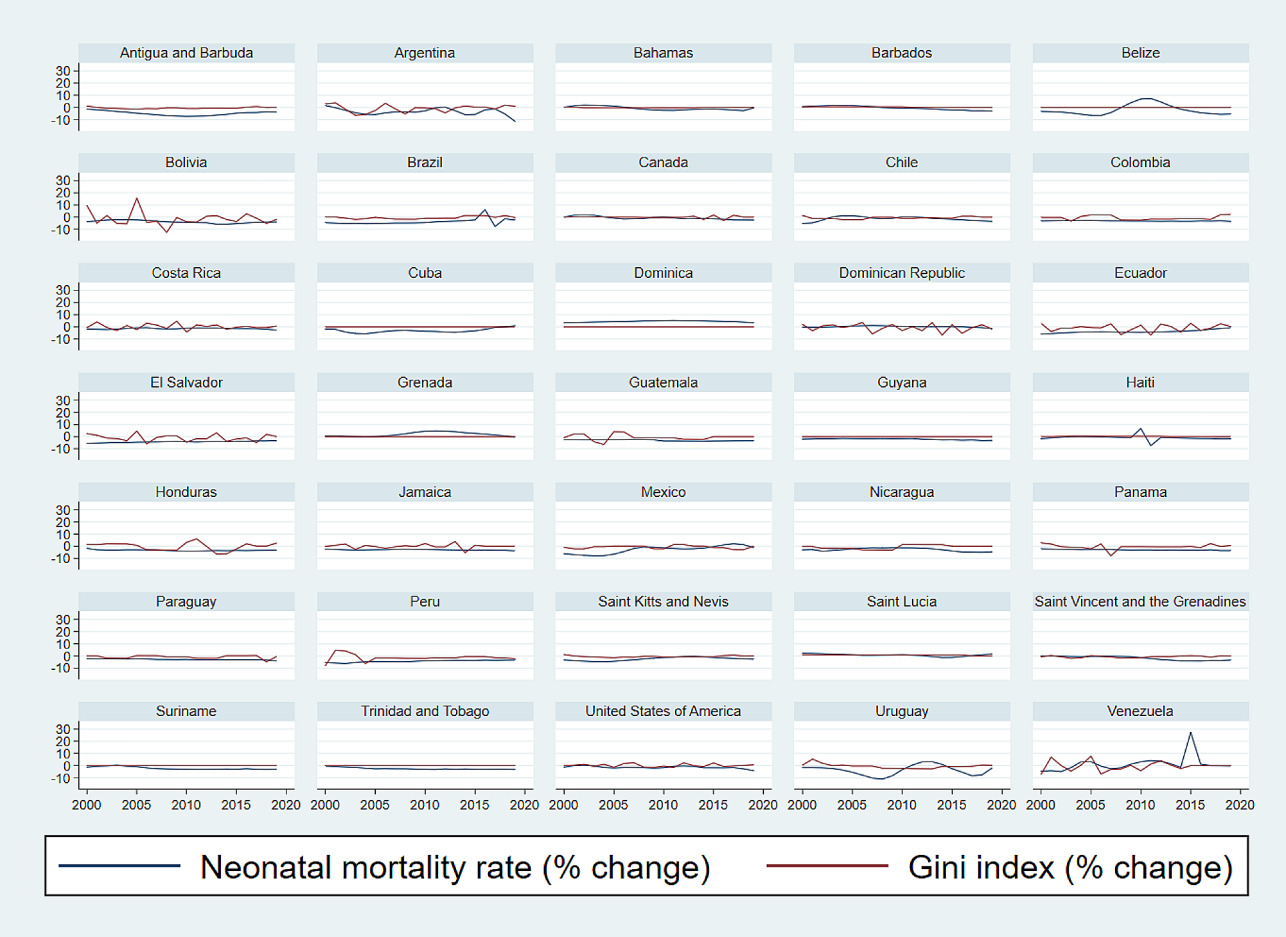
Neonatal mortality rate and Gini index percentage changes for countries of the Americas, 2000–2019

Figure 1 and Figure  2 give an insight into the time-dynamics of the neonatal mortality rate and the Gini index for the 35 countries. They illustrate trends in the two variables (Fig. 1) and their annual percentage change (Fig. 2). As illustrated in Fig. 1, the neonatal mortality rate and the Gini index have both decreased over time in most countries. In Bahamas, Canada, Costa Rica, Grenada, and the United States, the neonatal mortality rate and the Gini index have remained relatively stable over time (Fig. 1). In Dominica, Grenada, and Saint Lucia there have been some increasing trends in these variables (Fig. 1). The annual percentage change in these variables has mostly been negative and its absolute value lower than 10% (Fig. 2)

**Fig. 3:**
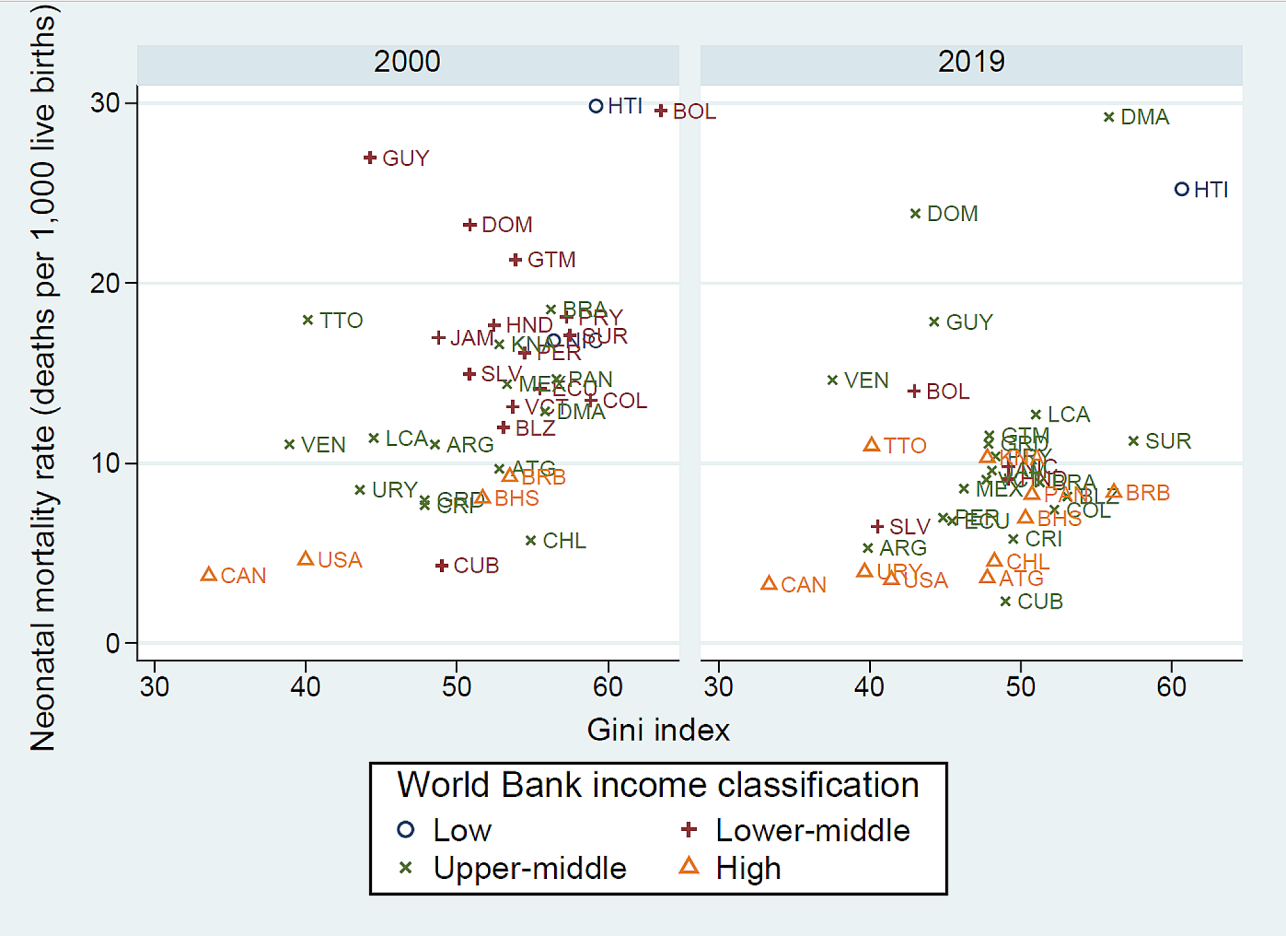
Associations between the Gini index and the neonatal mortality rate for countries of the Americas, 2000 and 2019

Figure 3 presents scatterplots to visualize preliminary cross-sectional associations between the Gini index and the neonatal mortality rate in the Americas during 2000 and 2019. As can be seen, there is a positive relationship between both variables. This positive relationship is quantified by Pearson correlation coefficients of 0.47 (*P* =.004) in 2000 and 0.33 (*P* =.05) in 2019. Furthermore, Ordinary Least Squares estimates for regressions of the neonatal mortality rate on the Gini index in 2000 and 2019 are 0.48 (95% CI [0.16, 0.79], *P* =.004) and 0.35 (95% CI [−0.001, 0.70], *P* =.05), respectively. Figure 3 also illustrates that higher-income countries tend to have lower neonatal mortality rates than lower-income countries

### Income inequality as a determinant of neonatal mortality in the Americas, 2000–2019

The results for the regression models estimated by G2SLS that quantify the relationship between the neonatal mortality rate and the Gini index are reported in Table 2. Model (1) is a core specification which includes the Gini index as the sole regressor. The result implies that a unit increase in the Gini index is associated with a 0.50 (95% CI [0.27, 0.73], *P* =.00) increase in the neonatal mortality rate. Model (2) extends the core specification by including logged GDP per capita, health expenditure as percentage of GDP, and the secondary school enrollment rate as socioeconomic and macroeconomic control variables. The result suggests that a unit increase in the Gini index is associated with a 0.50 (95% CI [0.26, 0.74], *P* =.00) increase in the neonatal mortality rate.

Specification (3) includes the percentage of newborns with low birth weight as a control variable related to neonates and their health and also controls for logged GDP per capita. The coefficient estimate for the Gini index indicates that a unit increase in it is associated with a 0.27 (95% CI [0.02, 0.52], *P* =.03) increase in the neonatal mortality rate. In specification (4), besides logged GDP per capita, we also control for factors related to national health systems (the percentage of births attended by skilled health personnel and the percentage of family planning satisfied with modern methods). The results indicate that a unit increase in the Gini index is associated with a 0.28 (95% CI [0.09, 0.46], *P* =.00) increase in the neonatal mortality rate. Finally, specification (5) includes all of the control variables, namely logged GDP per capita, health expenditure as a percentage of GDP, the secondary school enrollment rate, the percentage of newborns with low birth weight, the percentage of births attended by skilled health personnel, and the percentage of births attended by skilled health personnel. The estimates in this specification suggest that a unit increase in the Gini index is associated with a 0.27 (95% CI [− 0.04, 0.57], *P* =.09) increase in the neonatal mortality rate.

**Table 2:** Regression results for the relationship between neonatal mortality and the Gini index

			Model		
Variable	(1)	(2)	(3)	(4)	(5)
Gini index	0.50	0.50	0.27	0.28	0.27
	[0.27, 0.73]	[0.26, 0.74]	[0.02, 0.52]	[0.09, 0.46]	[− 0.04, 0.57]
	(0.00)	(0.00)	(0.03)	(0.00)	(0.09)
Logged GDP per capita (PPP)		−1.08	−1.39	−1.62	−2.01
		[− 1.84, − 0.32]	[− 2.78, − 0.00]	[− 2.19, − 1.04]	[− 3.03, − 1.00]
		(0.01)	(0.05)	(0.00)	(0.00)
Health expenditure (% of GDP)		−0.41			−0.37
		[− 0.77, − 0.05]			[− 0.81, 0.06]
		(0.03)			(0.09)
Secondary school enrollment (%)		0.01			0.07
		[− 0.07, 0.10]			[0.01, 0.13]
		(0.79)			(0.02)
Low birth weight among newborns (%)			1.39		0.89
			[0.16, 2.62]		[0.16, 1.62]
			(0.03)		(0.02)
Skilled attendant at birth (%)				−0.07	−0.09
				[− 0.12, − 0.02]	[− 0.14, − 0.03]
				(0.01)	(0.00)
Family planning satisfied with modern methods (%)				−0.09	−0.06
				[− 0.26, 0.08]	[− 0.21, 0.09]
				(0.31)	(0.44)
Observations	700	404	432	447	200
Countries	35	33	27	32	27
Sargan–Hansen statistic	0.19	0.27	0.55	0.13	0.29

*Note* The dependent variable is the neonatal mortality rate as measured by the number of neonatal deaths per 1,000 live births. Equations are estimated by G2SLS with lags two to four of the Gini index used as its instrumental variables and cluster-robust standard errors. All specifications include country random effects and year fixed effects. *P*-values are reported in parentheses and 95% confidence intervals are presented in square brackets. Reported Sargan–Hansen statistics are *P*-values

### Implications of changes in income inequality during the 2020–2030 period to achieve the SDG target for the neonatal mortality rate by 2030

Table 3 presents the implications of changes in the Gini index during the 2020–2030 period to achieve the SDG target for the neonatal mortality rate by 2030. The analysis is performed using the median coefficient estimate for the neonatal mortality rate across all models in Table 2, which corresponds to model (5). As of 2019, the countries in the Americas that have a neonatal mortality rate higher than 12 neonatal deaths per 1,000 live births (the SDG 3.2 target for 2030) are: Bolivia (14.01), Dominica (29.23), the Dominican Republic (23.87), Guyana (17.86), Haiti (25.23), Saint Lucia (12.7) and Venezuela (14.62). Hence, the minimal reductions in the neonatal mortality rate during the 2020–2030 period to achieve the SDG 3.2 target in these countries are: 2.01 (Bolivia), 17.23 (Dominica), 11.87 (the Dominican Republic), 5.86 (Guyana), 13.23 (Haiti), 0.7 (Saint Lucia), and 2.62 (Venezuela). Based on the predictions of our regression model (5) in Table 2, and keeping all other factors that influence the neonatal mortality rate fixed, the SDG 3.2 target in these countries may be achieved by a reduction in the Gini index during the 2020–2030 period of around 7.57 in Bolivia, 64.89 in Dominica, 44.7 in the Dominican Republic, 22.07 in Guyana, 49.83 in Haiti, 2.64 in Saint Lucia, and 9.87 in Venezuela.

**Table 3:** Predicted reduction in the Gini index for 2020–2030 to achieve the SDG 3.2 target for neonatal mortality

Country	Neonatal mortality rate in 2019	Minimal change in the neonatal mortality rate during 2020–2030 to achieve the SDG 3.2 target		Predicted change in the Gini index during 2020–2030 to achieve SDG 3.2	Minimum and maximum values of the Gini index during 2000–2019	Expected change in the Gini index during 2020–2030	
Bolivia	14.01		−2.01	−7.57 [− 16.21, 1.07]	42.96, 63.51	−8.26	
Dominica	29.23		−17.23	−64.89 [− 138.98, 9.2]	55.84, 55.84	.	
Dominican Republic	23.87		−11.87	−44.7 [− 95.75, 6.34]	43.03, 52.34	−3.78	
Guyana	17.86		−5.86	−22.07 [− 47.27, 3.13]	44.27, 44.27	.	
Haiti	25.23		−13.23	−49.83 [− 106.72, 7.06]	59.21, 60.66	1.45	
Saint Lucia	12.7		−0.7	−2.64 [− 5.65, 0.37]	44.5, 50.99	4.47	
Venezuela	14.62		−2.62	−9.87 [− 21.13, 1.4]	36.32, 43.08	−3.18	

^a^Reported predictions are means and are based on model (5) from Table 2.Their corresponding 95% confidence intervals are in square brackets

^b^ The expected change in the Gini index during 2020–2030 is based on 2000–2019 trends. No trend data is available for Dominica nor Guyana

## Discussion

This study has estimated the relationship between income inequality, measured by the Gini index, and the neonatal mortality rate, as measured by neonatal deaths per 1,000 live births, in 35 countries in the Americas during 2000–2019. The results suggest a positive relationship between the Gini index and the neonatal mortality rate. Based on the results, a unit increase in a country’s Gini index during 2000–2019 is associated with a 0.27 (95% CI [− 0.04, 0.57], *P* =.09) increase in the neonatal mortality rate.

Given the positive association between the Gini index and neonatal mortality, we estimated the reductions in the former during the 2020–2030 period that our analysis predicts would result in achieving the SDG 3.2 target of at least as low as 12 neonatal deaths per 1,000 live births by 2030. This was done for Bolivia, Dominica, the Dominican Republic, Guyana, Haiti, Saint Lucia and Venezuela, which are countries in the Americas that, as of 2019, have more than 12 neonatal deaths per 1,000 live births. The minimal reductions in the neonatal mortality rate during the 2020–2030 period to achieve the SDG 3.2 target for these countries were predicted to be achieved by reductions in their Gini index of 7.57 in Bolivia, 64.89 in Dominica, 44.7 in the Dominican Republic, 22.07 in Guyana, 49.83 in Haiti, 2.64 in Saint Lucia, and 9.87 in Venezuela.

If trends in the Gini index for 2000–2019 are maintained in these countries, during 2020–2030 we would expect changes in the Gini index of − 8.26 in Bolivia, − 3.78 in the Dominican Republic, 1.45 in Haiti, 4.47 in Saint Lucia, and − 3.18 in Venezuela. Hence, our analysis suggests it may be plausible for Bolivia to achieve the SDG 3.2 target for neonatal mortality solely based on the expected reductions in income inequality during 2020–2030. Although the Gini index in the Dominican Republic and Venezuela has shown a downward trend during 2000–2019, our analysis predicts that even greater reductions in the Gini index than those expected would be required during 2020–2030 to achieve SDG 3.2 exclusively based on changes in income inequality. Finally, the analysis suggests that if rising trends in the Gini index during the last two decades continue in Haiti and Saint Lucia, progress towards achieving SDG target 3.2 in these countries could potentially be hindered. Altogether, considering Gini values and trends during 2000–2019 and the modest association between the Gini index and the neonatal mortality rate, these findings suggest a potential limited scope for income and wealth redistribution policies to support reductions in neonatal mortality in countries in the Americas. Thus, policies and interventions to ensure higher coverage and quality of services provided by national health systems, as well as to reduce socio-economic inequalities in health, remain essential to end preventable neonatal deaths in the region.

One of the limitations of this study is the limited availability of data for control variables, which reduces the number of observations in models in which they are included and may difficult interpreting the results as applicable to all 35 countries over the entire time period studied. Another limitation is its key assumption of keeping all other factors that influence the neonatal mortality fixed when inferring country-level changes in income inequality during the 2019 to 2030 period to achieve SDG 3.2. Not only is this implausible in a normal policy setting, but it also overlooks other determinants of neonatal mortality. Moreover, these predictions are based on extrapolating the estimated relationship between the Gini index and the neonatal mortality rate to ranges in the Gini index not observed in the countries during the period studied. However, the estimated linear association may not persist outside of the observed ranges in the Gini index nor during the years 2020–2030. Furthermore, due to the unavailability of data after 2019, the analysis does not consider possible effects of the COVID-19 pandemic on changing trends in neonatal mortality, income inequality or changing the relationship of income inequality as a determinant of neonatal mortality. Progress in reducing neonatal mortality in the Americas may be hindered by the COVID-19 pandemic [25]. Nonetheless, there is currently no evidence of significant changes in neonatal mortality in the Americas during 2020 [26]. Another limitation of this study is that no other income inequality measure is analyzed besides the Gini index, so it is important to consider its shortcomings as a measure of income inequality [27].

Progress in reducing neonatal mortality in the Americas has been at a slower rate than the progress achieved in reducing under-5 mortality [28]. Consequently, the proportion of under-5 deaths during the neonatal period has been increasing during the last decades in the Americas region [1, 29]. Currently, 55% of under-5 deaths occur in the neonatal period in the Americas [1]. As a result, progress in reducing neonatal mortality would translate into progress in reducing under-5 mortality, promoting the overall achievement of the SDG 3.2 target in the region. This underscores the importance of addressing the factors that influence neonatal mortality. For instance, preterm birth complications, intrapartum-related events and infections including sepsis, meningitis and pneumonia have been found as the main proximate causes of neonatal deaths [30, 31]. Thus, it is important for policy makers to address these proximate causes. Improving our understanding of the underlying causes driving neonatal deaths is essential. This knowledge will provide invaluable insights and enable greater room for evidence-based policy making to end with preventable child deaths.

## Data Availability

Data can be made available for review upon reasonable request.
